# Addressing the gap in clinical research education: Implementation of the Introduction to Clinical Research Training—Japan program

**DOI:** 10.1002/jgf2.204

**Published:** 2018-09-15

**Authors:** Takuhiro Moromizato, Vanessa Garcia‐Larsen, Djøra Soeteman, Paige G. Wickner, Joaquim M. Havens, Rachel Lund, Rieko Eriguchi, Yasuharu Tokuda, Kenji Murata, Kunitoshi Iseki, Ajay K. Singh, Kenneth B. Christopher

**Affiliations:** ^1^ Internal Medicine Department Renal & Rheumatology Division at the Okinawa Nanbu Medical Center and Children's Medical Center Okinawa Japan; ^2^ Department of International Health Johns Hopkins Bloomberg School of Public Health Baltimore Maryland; ^3^ Center for Health Decision Science Harvard T.H. Chan School of Public Health Boston Massachusetts; ^4^ Division of Rheumatology, Immunology and Allergy Department of Quality and Safety Brigham and Women's Hospital Harvard Medical School Boston Massachusetts; ^5^ Division of Trauma, Burns and Surgical Critical Care Brigham and Women's Hospital Harvard Medical School Boston Massachusetts; ^6^ Postgraduate Medical Education Harvard Medical School Boston Massachusetts; ^7^ Nephrology Department Kaizuka Hospital Fukuoka City Japan; ^8^ Muribushi Okinawa for Teaching Hospitals Okinawa Japan; ^9^ The Okinawa Nanbu Medical Center and Children's Medical Center Okinawa Japan; ^10^ Clinical Research Support Center at Nakamura Clinic Okinawa Japan; ^11^ Harvard Medical School Boston Massachusetts; ^12^ Division of Renal Medicine, Brigham and Women's Hospital Faculty Director for Global Education Postgraduate Medical Education Harvard Medical School Boston Massachusetts

The traditional research strength in Japan is basic science.^1^ Of Japan's published scientific articles, 75% come from chemistry and physical sciences.^2^ Clinical research in Japan has mainly focused on outcome studies, and its productivity has decreased in comparison with that of basic and life sciences.^3^ Moreover, opportunities for international collaboration have been limited in Japan as evidenced by the relatively fewer international collaborative observational studies or clinical trials compared to most developed countries.^4^

For Japanese junior faculty and postdoctoral fellows, much time is devoted to providing clinical care during their most important stage of development as clinical researchers.^1^, ^5^ Survey results from Kyoto University Hospital indicate that Japanese physicians seek greater knowledge of clinical research design and biostatistical concepts.^6^ In Japan, only a few institutes offer formal clinical research training including Kyoto University, University of Tokyo, Keio University, and St. Luke's International Hospital. A small number of well‐supported Japanese clinicians relocate to other countries for 1‐2 years to seek clinical research training.^7^

To address the shortage of trained clinical researchers in Japan, we implemented a 6‐month blended learning certificate course in Japan in January 2018: the Introduction to Clinical Research Training (ICRT)—Japan program. In 2016‐2017, under the guidance of two Japanese clinical scientists, four Japanese learners enrolled in and completed the Introduction to Clinical Research Training course in Dubai administered by Harvard Medical School. Based on feedback from these Japanese learners, the curriculum was tailored to a Japanese clinical audience and the Introduction to Clinical Research Training course was implemented in Japan by Postgraduate Medical Education at Harvard Medical School. The program objectives are presented in Table 1. This custom‐built course was characterized by a blended learning model with 33 recorded online lectures, 10 live interactive webinars, two team assignments, and an individual final project, bookended by two intense 4‐day workshops at the Okinawa Institute of Science and Technology. The workshop, recorded online and webinar faculty, was comprised of three Japanese clinical scientists and twenty clinical scientists from Harvard Medical School, the Harvard T.H. Chan School of Public Health and The Johns Hopkins Bloomberg School of Public Health.

**Table 1:** ICRT‐Japan program objectives

Demonstrate a clear understanding of the core concepts of biostatistics and epidemiology
Develop a research question and formulate a testable hypothesis
Apply the design, implementation, and presentation of a clinical research study
Write an organized and structured manuscript
Critically evaluate medical literature
Synthesize essential statistical analyses using STATA software

The novel curriculum included training in epidemiology, biostatistics, statistical programming, scientific writing, data visualization techniques, ethics, cost‐effectiveness analysis, decision science, meta‐analysis, systematic reviews, research leadership concepts as well as the design, implementation, and reporting of clinical trials. The team assignments included a “pitch‐our‐study” and a consent form evaluation for which teams of nine learners had to work together and present live to Harvard faculty via webinar. The individual final assignment required learners to design, implement, and analyze a study using an existing training dataset. This required skills in statistical computing, writing a 300‐word abstract, and the recording of an individual 3‐minute project presentation reviewed by peers and Harvard faculty using the Practice application (https://www.practice.xyz). The Harvard Medical School Canvas Learning Management System was used to distribute the online course content to learners and is specifically designed for quizzing, assignment generation, collaboration, and learner engagement and assessment. An overview of the full curriculum and faculty can be found online (https://postgraduateeducation.hms.harvard.edu/certificate-programs/open-enrollment-programs/introduction-clinical-research-japan).

Course tuition (US$ 6000/learner) was partially financed by the Okinawa Asia Clinical Investigation Synergy (OAICS), a nongovernmental organization formed by a coalition of entities including Okinawa Prefectural Government, University of the Ryukyus, Okinawa Prefectural Hospital Group, Muribushi Residency Hospitals, Okinawa Medical Association, Okinawa Institute of Science and Technology, Healthcare institutes in Okinawa, and other Japanese universities and hospitals. OAICS was founded by Dr. Kunitoshi Iseki and is currently under his spirited leadership.

OAICS leadership worked closely with its member entities to identify 40 Japanese learners whose applications were reviewed by the Harvard Medical School for course suitability. Two other Japanese learners applied following Japanese scientific association announcements. Applicants were required to have an affinity for clinical research and an MBBS, MD, PhD, DMD, PharmD, or equivalent degree. Marketing by Harvard Medical School resulted in an additional 10 learners from outside Japan enrolling in the course. Fifty‐two learners enrolled in the program from nine countries. Forty‐two learners came from Japan, two each from the Philippines and China, and one each from Australia, Israel, Myanmar, South Korea, Taiwan, and the United States. As the ICRT‐Japan program is designed for working adults, the academic course commitments were scheduled so as not to interfere with learners’ current training programs. The mean time learners spent engaged with the Canvas course website was 5.6 hours per week.

The course culminates in receiving a certificate of completion for the ICRT‐Japan program, which is generally regarded as an asset on the curriculum vitae both nationally and internationally. Academic standards to obtain the certificate include meeting all assignment deadlines, a pass on all nine quizzes, 75% webinar attendance, active participation in team assignments, completion of individual abstract and individual presentation, and full attendance of both in‐person workshops. Fifty learners completed the program within 6 months. Two learners were unable to attend the final workshop and deferred completion of the course to 2019.

Learner feedback after the first year of the ICRT‐Japan program was positive and will be used to fine‐tune the curriculum. To gain perspective into the student experience, we include the following remarks from our student speaker at graduation:

I believe the ICRT program was rich and productive for us. The classes were very understandable and the teachers were very kind and passionate. They even answered our questions outside of class time.

For me, the most memorable assignments were the team assignments. Our team meetings started at 10 pm after our daily work. We set a different chairman and timekeeper each meeting and shared our opinions openly. We realized the difficulty in uniting our different opinions within a limited time. This will lead to improvement of actual clinical studies and collaborative studies.

We all come from different fields and various areas of medicine. We can make the most of what we have learned for our clinical study. It is my hope that we will keep in touch and develop the relationships that were started here. Graduation isn't the end of our tough journey, but a beautiful beginning.

Rieko Eriguchi, MD, PhD

It is too early to determine the long‐term benefits of the ICRT‐Japan program in terms of publishing clinical research manuscripts and obtaining research funding. ICRT graduates will be surveyed annually to track such long‐term benefits. Immediate benefits to ICRT graduates are the clinical research skill set gained, a national and international network of fellow learners including those of other programs from Postgraduate Medical Education at Harvard Medical School, the Associate Alumni status at Harvard Medical School, and access to other courses offered by Harvard Medical School to promote further education. To illustrate this, four Japanese graduates of the ICRT program are current students in the advanced year‐long Global Scholar Clinical Research Training program and one Japanese clinical research student is enrolled in the 2‐year Masters in Medical Science program both offered by Postgraduate Medical Education at Harvard Medical School.

In conclusion, the ICRT‐Japan program provides rigorous, high‐quality, and affordable training aimed to address current gaps in clinical research education in Japan. The training of clinical research investigators is an important step toward ultimately increasing clinical research collaborations and productivity in Japan.

## CONFLICT OF INTEREST

The authors have stated explicitly that there is no conflict of interest in connection with this article.
